# Structure and ion physiology of *Brasenia schreberi* glandular trichomes in vivo

**DOI:** 10.7717/peerj.7288

**Published:** 2019-07-10

**Authors:** Chaodong Yang, Xia Zhang, Fan Zhang, Xiaoe Wang, Qingfeng Wang

**Affiliations:** 1 The School of Horticulture and Gardening, Yangtze University, JIngzhou, Hubei, China; 2 Wuhan Botanical Garden, The Chinese Academy of Sciences, Wuhan, China

**Keywords:** *Brasenia schreberi*, Glandular trichome, Ethanol stress, Structure, Histochemistry, Ionic permeability, NaCl stress

## Abstract

*Brasenia schreberi* is a critically endangered aquatic basal angiosperm. In this work, we characterized the structure of the glandular trichomes of *B. schreberi* morphologically and histochemically. We used a variety of structural, histochemical and permeability stains for the characterization, and we tested the effects of stress in vivo using NaCl and ethanol. We observed that the glandular trichome of *B. schreberi* are composed of two disk-like stalk cells, and a glandular cell which surround a cuticular storage space. The cuticle is discontinuous at the surface of the shoots. Nearly half of young trichomes senesced in 0.9% NaCl, and mature trichomes senesced at 1.8% NaCl. About half of young trichomes senesced under 3% ethanol and mature trichomes senesced in 2% ethanol after 20 min of treatment. The physiology of glandular trichomes affects the way they secrete mucilage via storage space at a young stage. The trichomes become permeable and absorb ions when mature. This transition depends on the osmiophilic material and the dynamic protoplast. It can accelerate senescence and disassembly by ion accumulation. Permeability tests and ion treatments of glandular trichomes provide new insights for fertilizer research. Our study highlights the structure and physiology of *B. schreberi* glandular trichomes.

## Introduction

The water-shield, *Brasenia schreberi* J. F. Gmel. (Cabombaceae, Nymphaeales), is a well-known basal angiosperm plant (Williamson & Schneider, 1993; The Angiosperm Phylogeny Group, 2016). In China, it is listed as endangered as a result of habitat loss (Fu & Wiersema, 2001; Zhang et al., 2015). It is eaten as a vegetable and used as a medicinal plant. Its compounds have anti-inflammatory effects and can inhibit HIV-1 reverse transcriptase and viral replication (Legault et al., 2011; Hisayoshi et al., 2015). The gross morphology of the plant consists of vertical and horizontal rhizomes, floating stolons with peduncles, and adventitious roots (Moseley et al., 1984; Williamson & Schneider, 1993). The shoots are covered on the surface with hydrophilic lubrication mucilage (Wilkinson, 1979; Liu et al., 2014). Some of its anatomical features have been thoroughly studied, including the adventitious roots (Seago, 2002; Seago et al., 2005), stems with leaves (Xu et al., 1999), internal primary xylem (Schneider & Carlquist, 1996), and surface trichomes or mucilaginous hairs (Schrenk, 1888; Keller, 1893; Wang, 1989; Shi et al., 1991; Carpenter, 2006).

Glandular trichomes are epidermal protuberances composed of basal cells, stalk cells and trichome head cells. Each also has a cuticular storage space for secreted metabolites, such as terpenoids, essential oils and polysaccharides, though the exact contents vary across species and taxonomic groups (Carpenter, 2006; Bergau et al., 2015; Huchelmann, Boutry & Hachez, 2017; Tissier, Morgan & Dudareva, 2017). However, the glandular trichomes of *B. schreberi* have been reported to have one or two head cells on shoots (Schrenk, 1888; Keller, 1893; Zhou & Lu, 1985; Shi et al., 1991; Carpenter, 2006).

Many studies have been conducted on the stress caused by heavy metals (e.g., As^3+^, Cd^2+^, Cr^6+^, Hg^2+^) on *B. schreberi* winter buds and leaves that adversely affect the structure of membranes (Ding et al., 1998; Li et al., 1998; Shi et al., 2000; Yang et al., 2000; Yang, Shi & Chen, 2001), and decrease the activity of protective enzymes (Song, Shi & Yang, 2000; Xu, Liu & Shi, 2000; Xu et al., 2000; Yang et al., 2001), biochemical properties and photosynthesis (Chen et al., 1999; Lu et al., 1999). Small farmers pour solid fertilizer (about 50 kg) and pesticide (about 500 g) into the water of fields, and eyewitnesses have found *B. schreberi* leaves rot away in a few days (Zhang et al., 2015).

In this study, our aim was to investigate the anatomical and histochemical features of glandular trichomes, which have not been adequately examined in previous studies. We hypothesize that glandular trichomes can be damaged by large amounts of solid fertilizer and pesticide. We used NaCl and ethanol to represent all kinds of fertilizer and pesticide in order to imitate small farming operations (Fu & Wiersema, 2001; Zhang et al., 2015). Here, we analyze the anatomy and histochemistry, including ionic permeability, of the epidermal glandular trichomes of *B. schreberi* under brightfield, fluorescence, confocal, and transmission electron microscopy, especially with respect to the short-term effects of NaCl and ethanol, which are associated with accelerated degeneration and senescence in the trichomes.

## Materials and Methods

### Plant sourcing and collection

*Brasenia schreberi* specimens were collected during their growing season in late spring. Whole fresh plants were collected from Jichang Agricultural Development Ltd., Lichuan, Hubei Province, China. Using a two-edged blade under a stereoscope, we sectioned young, mature, and aged (or senescent) stems and leaves, and preserved the specimens in formaldehyde–acetic acid–alcohol for samples identification.

### Microstructure, histochemistry, and ionic permeability

Sudan red 7B (0.1%) dissolved in polyethylene glycol (average mw 400 Daltons) and 90% glycerol (v/v) was used to stain cuticles (Brundrett, Kendrick & Peterson, 1991; Yang et al., 2019; Zhang, Yang & Seago, 2018). Phloroglucinol–HCl was used to stain lignin (Yang et al., 2019; Zhang et al., 2017b; Zhang, Yang & Seago, 2018). To test ionic permeability, we used 0.05% and 0.1% berberine hemisulfate and 0.5% aniline blue to stain cuticles and membranes and the glandular trichomes (Brundrett, Enstone & Peterson, 1988; Seago et al., 1999; Yang et al., 2014; Zhang, Yang & Seago, 2018). These sections were observed and photographed using a Leica DME, Olympus IX71 for brightfield and epifluorescence microscopy.

We used 2.5 μg/mL Hoechst 33342 to stain nuclei for 10 min with 350 nm excitation and detected it at 455–465 nm (Pursel et al., 1985). These sections were observed and photographed using a Leica-SP8 confocal microscopy (Leica Camera, Wetzlar, Germany).

### Ultrastructure of transmission electron microscopy

Sections of 2 × 2 × 1 mm were fixed for 3 h in 2.5% glutaraldehyde, post-fixed in buffered 1% osmium tetroxide, and then dehydrated through an ethanol series. The samples were then embedded in Epon 812 resin. Ultrathin sections were placed on copper grids and stained with uranyl acetate followed by lead citrate and examined with a JEX-1400 transmission electron microscopy (JEOL, Tokyo, Japan) (Guo & Liu, 2013; Wang et al., 2008).

### NaCl and ethanol treatments

Four concentrations of NaCl solutions (0%, 0.9%, 1.8%, 2.7% w/v) and five concentrations of ethanol (0%, 1%, 2%, 3%, 4% v/v) were used to stress glandular trichomes at young and mature stages with six replicates per treatment. Sections of fresh petioles with mucilage were immersed in the above NaCl and ethanol solutions for specific amounts of time: 0, 10, 20, and 30 min. There were 216 experimental sections. After treatment, plant sections were rinsed five times with deionized water, lightly blotted, and sections were mounted in deionized water to be viewed under blue light and then photographed. The numbers of senescent (or aged) trichomes with ball-like, pillar-like, foam-like, or deformed protoplasts were counted as aged.

### Statistical analyses

We quantified the number of senescent trichomes that were stressed by the different concentrations and timing of the NaCl and ethanol treatments in the young and mature samples. The senescence of glandular trichomes is expressed as percentages under various NaCl and ethanol concentrations for treatment of different durations in young and mature stages were first transformed based on square root arcsine to normalize the data and then subjected to ANOVA using SAS 9.1 software (SAS Institute, Cary, NC, USA) and the means with standard deviations were compared using Duncan’s multiple range test at the <5% level. Graphs were plotted using original non-transformed values. Data are means of 36 samples.

## Results

### Structure and histochemistry of glandular trichomes

**Figure 1:**
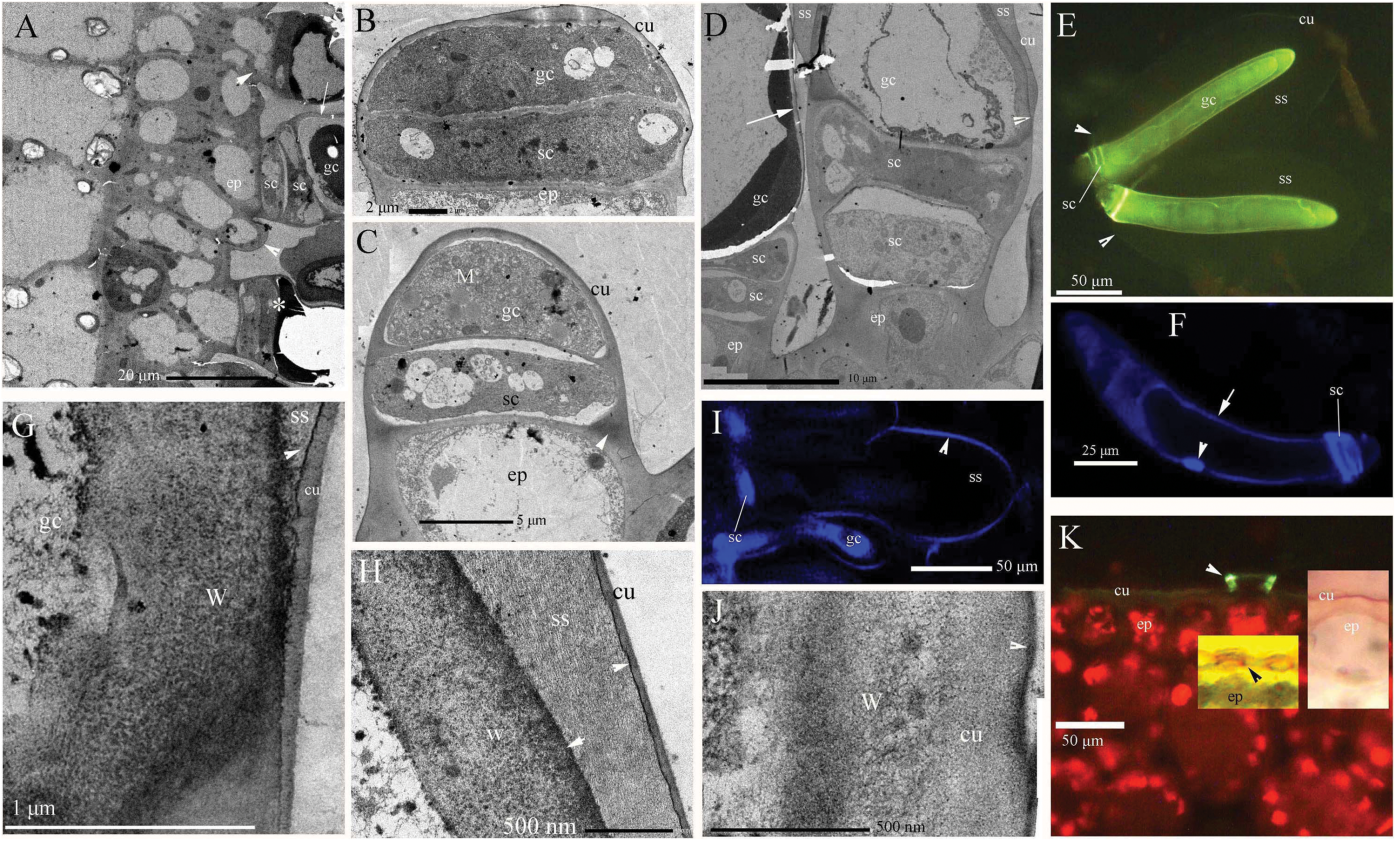
Photographs of micro- and ultra-structure and histochemistry of *B. schreberi* glandular trichomes. Scale bars on each plate. (A) Transverse sections of petioles; epidermis, and glandular trichomes (*), glandular trichome initially divided from the epidermis (lower arrowhead), the second stalk cell divided from epidermis before (upper arrowhead), cuticle on glandular cell (arrow), stalk cell, TEM; (B) glandular trichome divided into stalk cell and glandular cells, epidermis, cuticle, TEM; (C) glandular cell elongated with many mitochondria, stalk cell, lignin on the epidermis walls (arrowhead), cuticle, TEM; (D) glandular trichomes with storage space, and cuticle departed from the edge of the glandular cell to form storage space (arrowhead), stalk cell, epidermis, and cuticle (arrow), TEM; (E) glandular trichomes with whole storage space, cuticle connect to stalk cells (arrowhead), glandular cell, BAB; (F) single nucleus (arrowhead) in glandular cell, stalk cells, walls affinity to Hoechst 33342 (arrow), Hoechst 33342 stained, in vivo; (G) magnification of cuticle departing from glandular cell in D, walls and cuticle departing at beginning with osmiophilic material (arrowhead) in the middle, storage space, TEM; (H) magnification of the position in D (cu), large storage space between walls and cuticle, osmiophilic material (arrowhead), TEM; (I) surface of storage space affinity to Hoechst 33342, stalk cell, glandular cell, Hoechst 33342 stained, in vivo; (J) glandular cell walls after storage space sloughed off in A, D (arrow), osmiophilic material at surface (arrowhead), cuticle, cell walls, TEM; (K) base of glandular trichomes, lignified stalk cells (arrowhead), epidermal cuticle, BAB; middle inset shows lignified stalk cells (black arrowhead), Pg; right inset shows epidermal cuticle, SR7B, in vivo; (BAB, berberine hemisulfate–aniline blue; cu, cuticle; ep, epidermis; gc, glandular cell; M, mitochondria; Pg, phloroglucinol–HCl; SR7B, Sudan red 7B; sc, stalk cell; ss, storage space; TEM, transmission electron microscope; W, cell walls).

In *B. schreberi* stems, petioles and abaxial leaves, the glandular trichomes showed visible mucilage around their sides and tips (Fig. 1A; Figs. S1A–S1C). Glandular trichomes initially divide from epidermis (Fig. 1A), then differentiate into stalk cells and glandular cells (Fig. 1B). Then the glandular cell elongates with many mitochondria (Fig. 1C). The elongated glandular cell is one cell and it is surrounded by storage space (Figs. 1D–1F). The surface of the storage space has a thin cuticle that contains osmiophilic materials in its inner face with affinity for Hoechst 33342 (Figs. 1G–1I). The walls of the glandular cell have thin cuticle and osmiophilic materials, and affinity to Hoechst 33342 (Figs. 1A, 1D, 1F and 1J) in the mature stage after storage space sloughed off. At the base of glandular trichomes, stalk cells have a cuticle and lignin (Figs. 1C and 1K). The epidermis has a discontinuous cuticle that is like a ring around the base of glandular trichomes (Fig. 1K), but it does not cover the protruding glandular cells in aged samples. In summary, the glandular trichome of *B. schreberi* consists of two disk-like stalk cells and a glandular cell which surrounds a storage space covered with a thin cuticle.

### Ionic permeability of glandular trichomes

**Figure 2:**
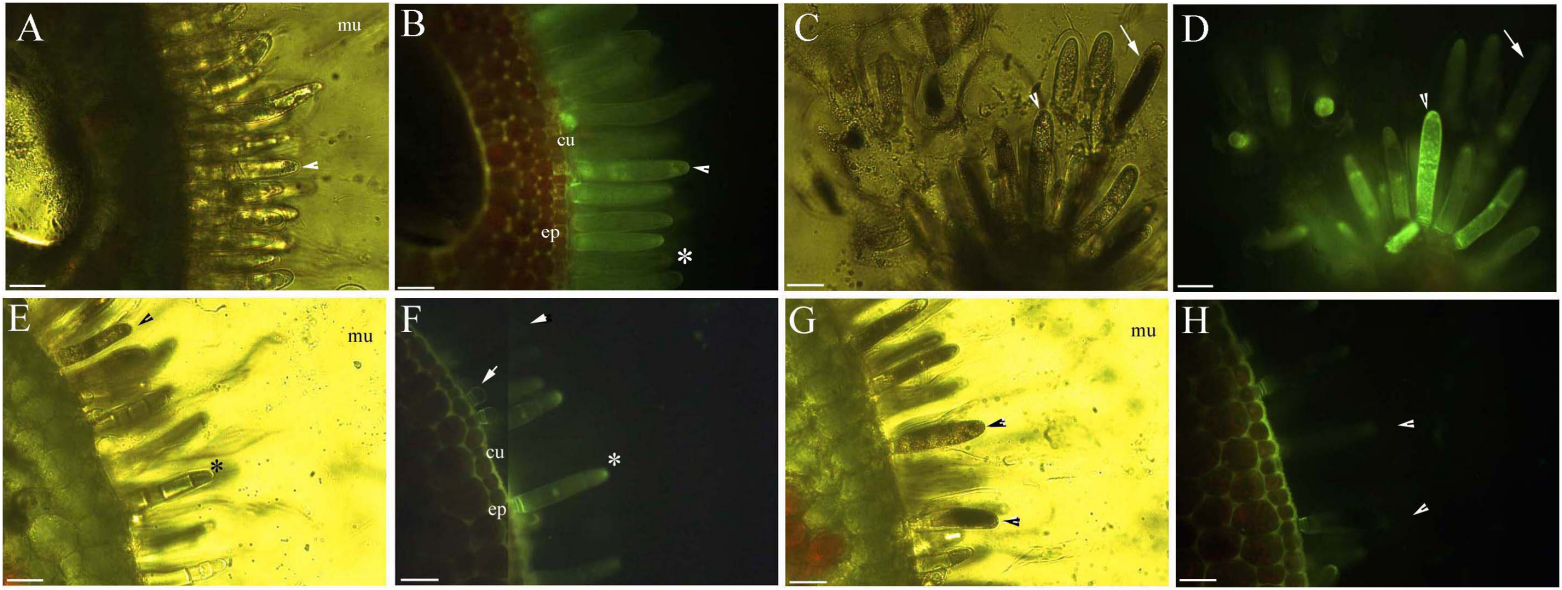
Photomicrographs of in vivo absorption of berberine by *B. schreberi* glandular trichomes. Scale bars = 50 μm. (A) Match to B; Glandular cells (arrowhead), mucilage, young stage, BAB under brightfield; (B) glandular cells absorb minimal berberine (arrowhead), glandular cells without berberine (asterisk), epidermis, cuticle, young stage, BAB; (C) match to D; Dots on mature stage glandular cells (arrowhead), black aged stage glandular cells (arrow), mature stage, BAB under brightfield; (D) mature glandular cells absorb berberine (arrowhead), aged glandular cells without berberine (arrow), mature stage, BAB; (E) match to F; Black aged glandular cells (arrowhead), pillar-like of glandular cells (asterisk), mucilage, aged stage, BAB under brightfield; (F) black aged glandular cells without berberine (arrowhead), pillar-like of glandular cells with minimal berberine (asterisk), small new emerge glandular cells without berberine (arrow), epidermis, cuticle, aged stage, BAB; (G) match to H; Black aged glandular cells (arrowhead), mucilage, aged stage, BAB under brightfield; (H) aged glandular cells without berberine (arrowhead), epidermis, cuticle, aged stage, BAB.

Young and aged glandular cells produced little or no fluorescence 0.5 s after staining, whereas mature glandular cells fluoresced intensely yellow seven to nine milliseconds after staining. Young glandular cells absorbed little to no berberine and thus fluoresced only slightly yellow (Figs. 2A and 2B), whereas mature glandular cells absorbed berberine and fluoresced intensely yellow (Figs. 2C and 2D). The oldest glandular cells absorbed little berberine and appeared black and pillar-like or lacked fluorescence (Figs. 2C–2H).

### Dynamic protoplast of glandular trichomes

**Figure 3:**
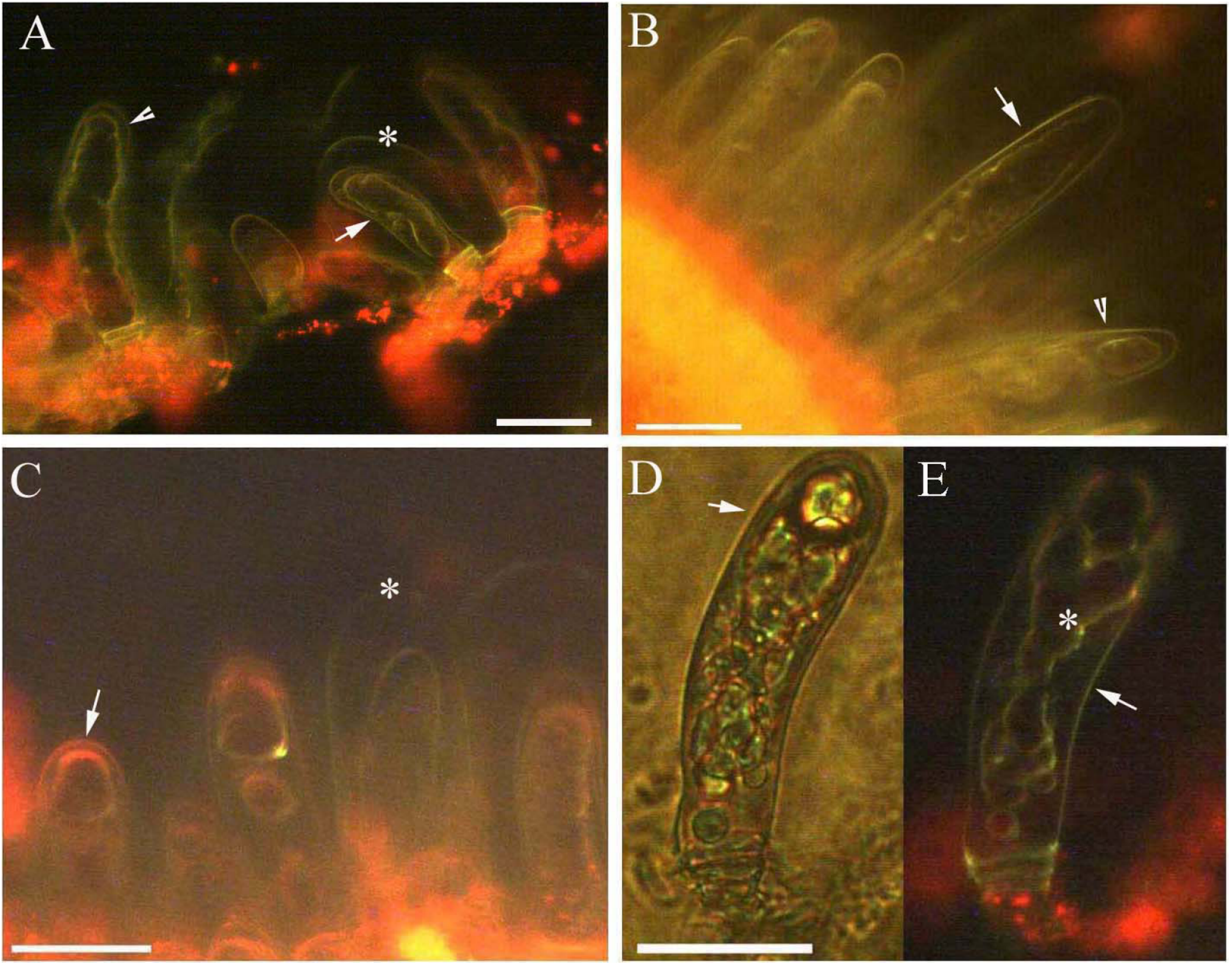
Photomicrographs of autofluorescence under blue excitation of *B. schreberi* glandular trichomes in vivo. Scale bars = 50 μm. (A) Storage space (asterisk), glandular cells (arrowhead), smoothness protoplast in glandular cells (arrow), young stage, non-stain; (B) glandular cells (arrowhead), spotty-like protoplast in glandular cells (arrow), mature stage, not stained; (C) storage space (asterisk), ball-like protoplast in glandular cells (arrow), aged stage, not stained; (D) large spotty-like protoplast in glandular cells (arrow), under brightfield; (E) glandular cells (arrow), large spotty-like and twisted protoplast (asterisk) in glandular cells, aged stage, not stained.

The protoplast of the glandular cells appeared smooth, spotted, and had short node- and pillar-like structures at young and mature stages when viewed under brightfield, epifluorescence and autofluorescence (Figs. 2B, 2D, 3A and 3B), or partly turned brown in color (Fig. 2E). The aged glandular cells turn black or have pillar-like, ball-like and twisted protoplast, and slough off with small new glandular cells, and some mucilage on stems and petioles (Figs. 2C, 2E–2H and 3C–3E).

### Effect of NaCl stress on glandular trichomes

**Figure 4:**
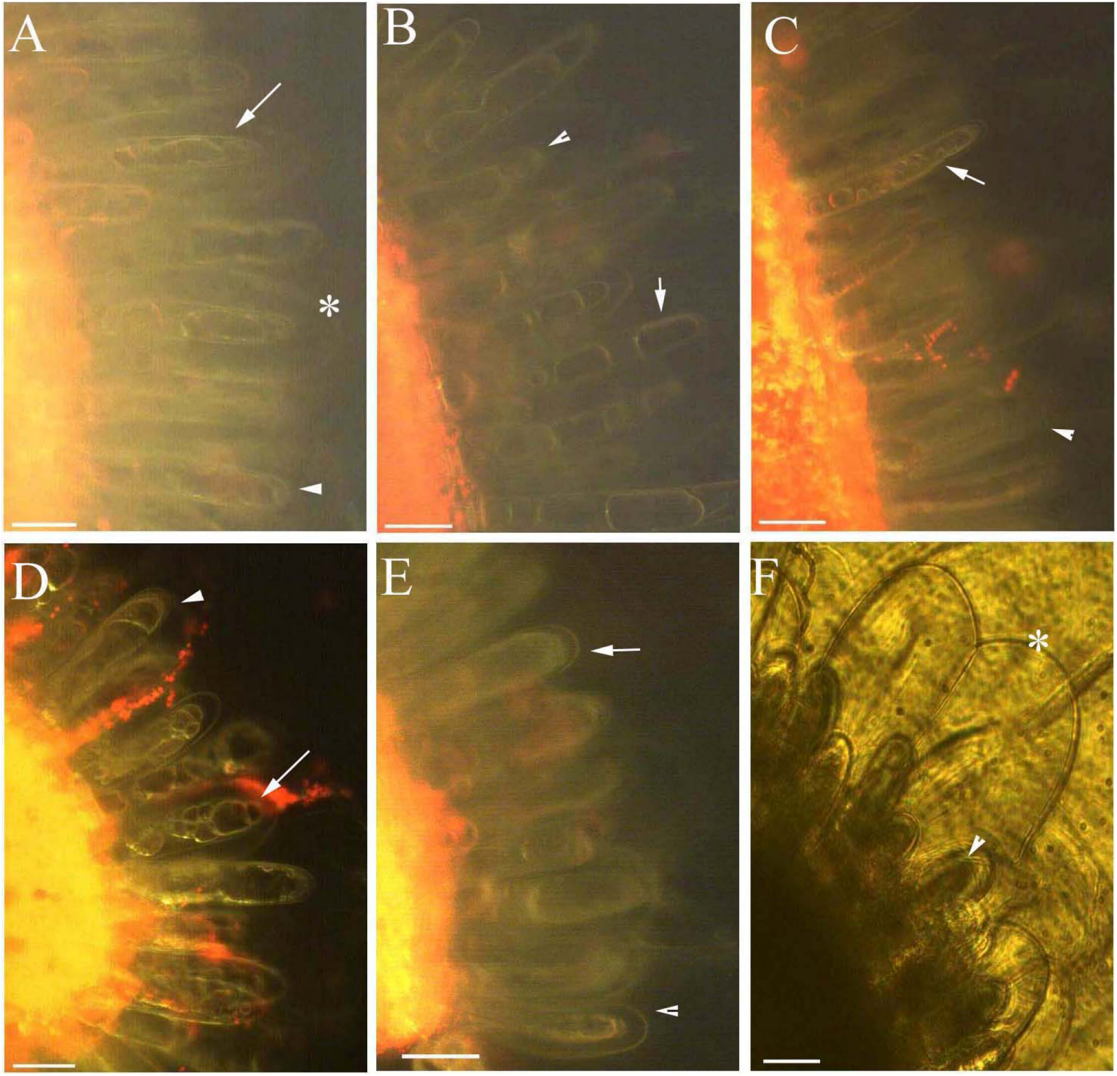
Photomicrographs of autofluorescence under blue excitation of *B. schreberi* glandular trichomes stressed by NaCl solutions in vivo. Scale bar = 50 μm. (A) Storage space (asterisk), glandular cells (arrowhead), smoothness protoplast (arrow), 0.9% NaCl and 10 min, young stage, not stained; (B) glandular cells (arrowhead), pillar-like protoplast in glandular cells (arrow), 0.9% NaCl and 30 min, young stage, not stained; (C) glandular cells (arrowhead), spotty-like protoplast in glandular cells (arrow), 1.8% NaCl and 10 min, mature stage, not stained; (D) glandular cells (arrowhead), more foam-like protoplast in glandular cells (arrow), 1.8% NaCl and 30 min, mature stage, not stained; (E) glandular cells (arrowhead), obscure protoplast in glandular cells (arrow), 2.7% NaCl and 20 min, mature stage, not stained; (F) storage space (asterisk), glandular cells (arrowhead), 2.7% NaCl and 10 min, mature stage, not stained.

**Figure 5:**
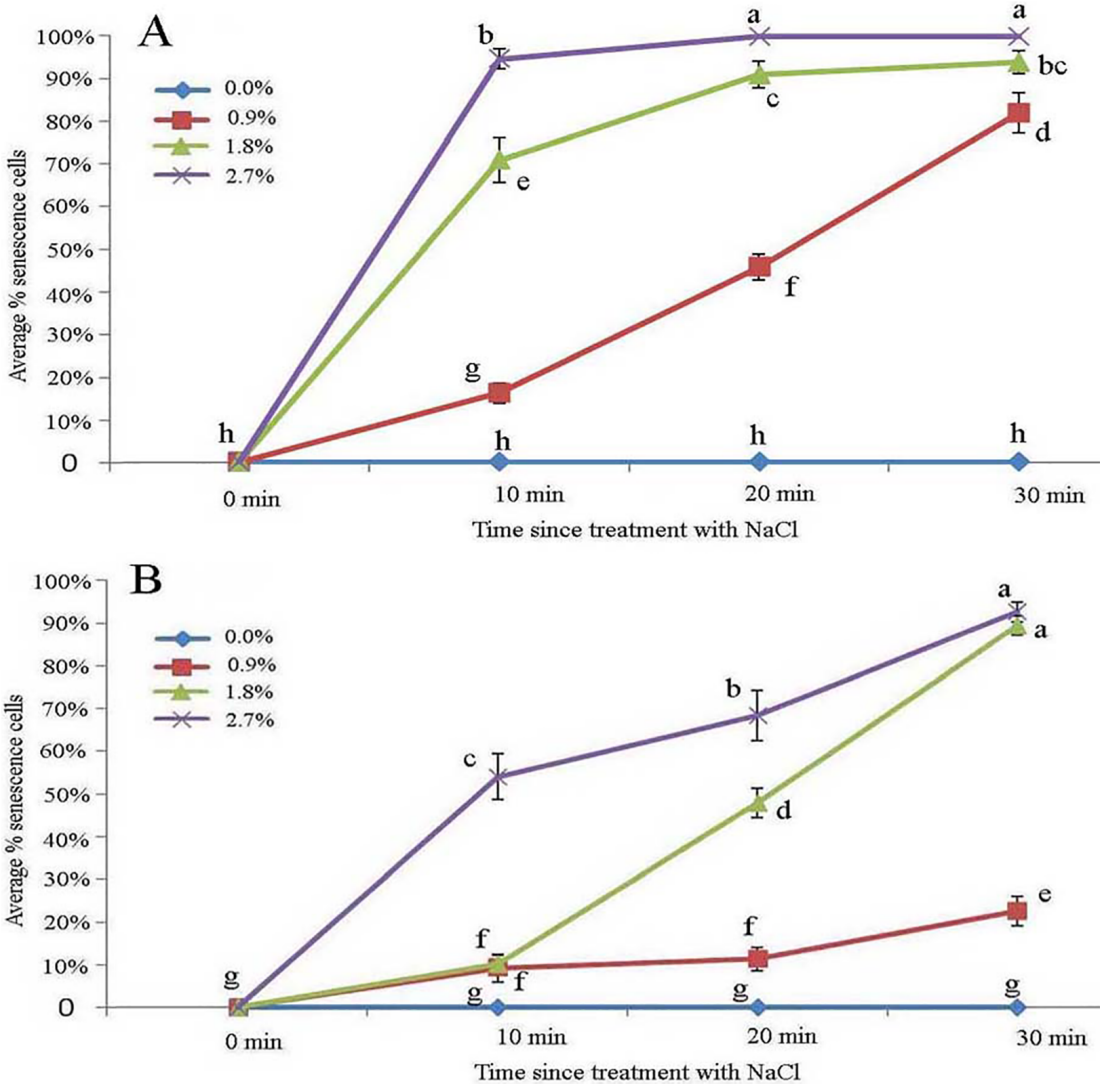
Average percentage of young (A) and mature (B) *B. schreberi* glandular cells that senesced after treatment with 0%, 0.9%, 1.8% or 2.7% NaCl after 0 min, 10 min, 20 min, and 30 min in vivo. Error bars represent 1 standard error of the mean (*n* = 6 replicates). The same letters are not significantly different according to Duncan’s multiple range test (*P* > 0.05).

Protoplasts appeared smooth in young glandular cells that were stressed by the 0.9% NaCl solution for 10 min (Fig. 4A). Almost half (45.8%) of the young glandular cells senesced after 20 min of treatment with 0.9% NaCl (Fig. 5A) and pillar-like structures were observed in aged glandular cells. Moreover, ca. 82.1% of all young glandular cells had pillar-like structures after 30 min of treatment with 0.9% NaCl (Figs. 4B and 5A). Mature glandular cells had spotted protoplasts after being stressed by 1.8% NaCl for 10 min (Fig. 4C). Almost half (47.9%) of senesced protoplasts formed a foam-like structure after 20 min of treatment of mature glandular cells with 1.8% NaCl (Fig. 5B), and approximately 89.5% of mature glandular cells had a foam-like protoplast after 30 min of treatment with 1.8% NaCl, which was significantly more than at 20 min post treatment with the same concentration of NaCl (Figs. 4D and 5B). More than half (54.0%) of mature senesced glandular cells had foam-structures and shrank or had heavily twisted protoplasts after being stressed by 2.7% NaCl for 10 min (Fig. 5B). We observed a disassembly of the protoplast in glandular cells when stressed by 2.7% NaCl for 20 min (Fig. 4E).

### Effect of ethanol stress on glandular trichomes

**Figure 6:**
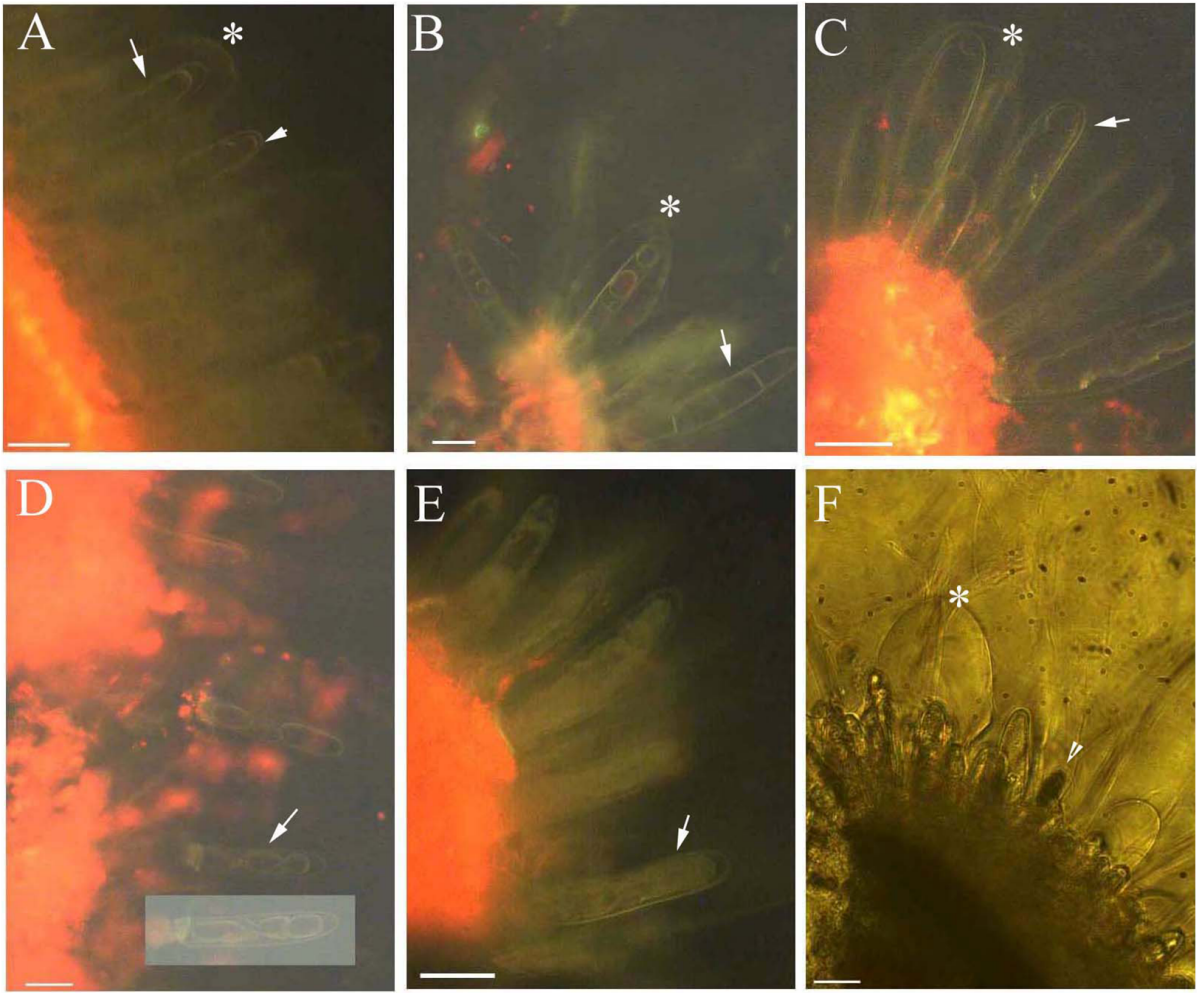
Photomicrographs of autofluorescence under blue excitation of *B. schreberi* glandular trichomes stressed by ethanol in vivo. Scale bars = 50 μm. (A) Storage space (asterisk), glandular cells (arrowhead), smoothness protoplast (arrow), 2% ethanol and 30 min, young stage, not stained; (B) storage space (asterisk), pillar-like protoplast in glandular cells (arrow), 3% ethanol and 30 min, young stage, not stained; (C) storage space (asterisk), smoothness protoplast (arrow), 1% ethanol and 20 min, mature stage, not stained; (D) foam-like protoplast in glandular cells (arrow), 2% ethanol and 30 min, mature stage, not stained; (E) obscure protoplast in glandular cells (arrow), 4% ethanol and 10 min, mature stage, not stained; (F) storage space (asterisk), glandular cells (arrowhead), 4% ethanol and 10 min, mature stage, not stained.

**Figure 7:**
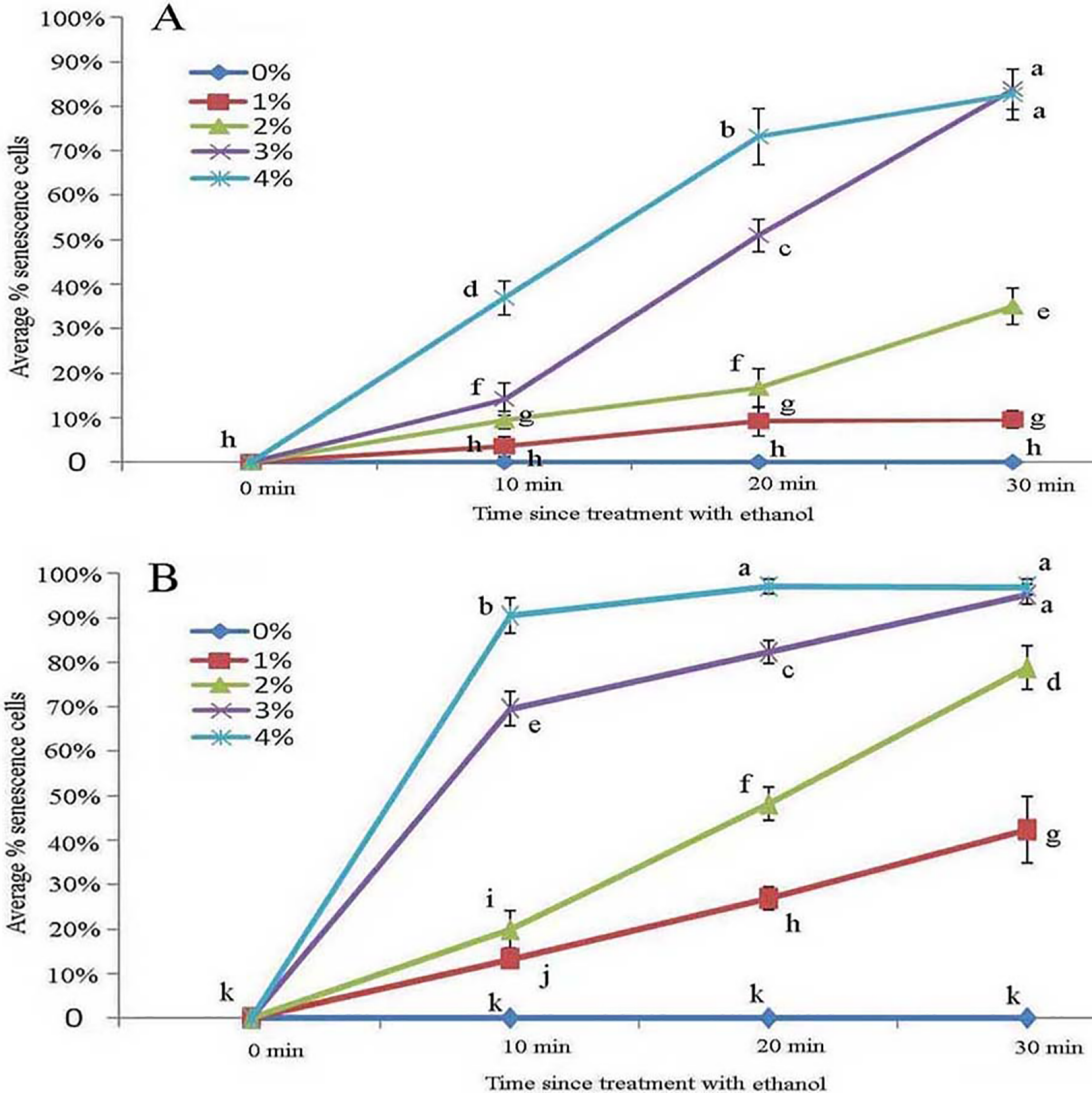
Average percentage of young (A) and mature (B) *B. schreberi* glandular cells that senesced after treatment with 0%, 1%, 2%, 3% or 4% (v/v) ethanol for 0 min, 10 min, 20 min, or 30 min in vivo. Error bars represent 1 standard error of the mean (*n* = 6 replicates). The same letters are not significantly different according to Duncan’s multiple range test (*P* > 0.05).

Across all ethanol treatments, we observed glandular cells senesced and disassembled compared with the control. The 1% ethanol treatment resulted in senescence of fewer glandular cells than in the untreated control group in the short term (0–10 min) among young glandular cells (Figs. 6A, 6B and 7A). When we looked at the effect of ethanol over time, we observed accelerated glandular cells senescence when stressed with 2%, 3% and 4% ethanol (Figs. 7A and 7B).

Protoplasts appeared smooth in young glandular cells stressed by 2% ethanol after 30 min with about one-third (35.1%) exhibiting senescence (Figs. 6A and 7A). In the 3% ethanol treatment, about half (51.2%) of the glandular cells that senesced had pillar-like structures after 20 min, and 84.2% had pillar-like structures after 30 min of treatment (Figs. 6B and 7A). In the mature glandular cells following 1% ethanol treatment, the protoplast had a smooth and spotty appearance and less than half (42.9%) senesced after 30 min of treatment (Figs. 6C and 7B). In the 2% ethanol treatment, almost half (48.8%) of mature glandular cells that senesced had foam-like protoplasts after 20 min and approximately 79.9% senesced glandular cells had foam-like protoplasts after 30 min of treatment (Figs. 6D and 7B). In the 3% ethanol treatment, most glandular cells that senesced (70.5%) exhibited a shrunken protoplast. In the 4% ethanol treatment, almost all (91.7%) protoplasts exhibited disassembly within 10 min (Figs. 6E and 7B). Both storage spaces of glandular trichomes were still present at all concentrations of NaCl and ethanol for treatments of durations (Figs. 4A, 4F, 6A–6C and 6F).

## Discussion

### Structure of glandular trichomes

In our study of the glandular trichomes of *B. schreberi*, we observed that these trichomes consist of two disk-like stalk cells and a glandular cell surrounding a storage space covered with a thin cuticle at the epidermal surface of stems, petioles, and leaves. Schrenk (1888) and Keller (1893) reported that glandular trichomes emerged from the epidermis and had two disk-like stalk cells and a glandular cell with one or two heads and surrounded a sac or storage space, and the glandular cell had three layers of cuticle (Zhou & Lu, 1985; Shi et al., 1991). In our study, we observed the glandular cell had a single layer of cuticle. The cuticle and subcuticle or cell walls cover the storage space surface of glandular trichomes, such as in *Artemisia annua* (Duke & Paul, 1993), Cajaninae (De Vargas et al., 2019), *Calceolaria volckmanni* (Sacchetti et al., 1996), *Cannabis sativa* (Mahlberg & Kim, 1991), *Cordia verbenacea* (Ventrella & Marinho, 2008), *Doronicum* (Muravnik, Kostina & Mosina, 2019), *Humulus lupulus* (Kim & Mahlberg, 2000), *Lippia citriodora* (Argyropoulou et al., 2010), peppermint (Turner, Gershenzon & Croteau, 2000), *Salvia argentea* (Baran, Özdemir & Aktaş, 2010), sunflowers (Amrehn et al., 2016), tobacco (Huchelmann, Boutry & Hachez, 2017), and tomatoes (Bergau et al., 2015). The protoplasts in glandular cells are smooth, spotted, and have short node- and pillar-like structures in the young and mature stages. In the aged stage, the protoplasts have pillar- or ball-like, structures and are twisted; they then turn black and slough off (Schrenk, 1888).

In our specimens, we did not find the very small structures termed hydropotes (Wilkinson, 1979) which Carpenter (2006) reported with illustrations in *B. schreberi*; instead, we find that the abaxial leaf surface and other shoot surfaces are densely covered with glandular trichomes that give the plant a very mucilaginous appearance. Wilkinson noted that the trichome-like structures produced by epidermal cells were hydropotes and were at first “mucilage-secreting hairs,” but later assumed the function of “absorbing water and mineral salts” (Wilkinson, 1979; Schrenk, 1888). While Carpenter (2006) explicitly stated that trichomes were lacking in *Brasenia*, Williamson & Schneider (1993) did report “occasional hydropote-like cells and numerous mucilage-secreting hairs” or trichomes. Ogden (1974) and Fahn (1990) reported such hairs in lacunae from New York, US, *B. schreberi*, but we did not observe glandular trichomes in aerenchymatous lacunae.

The glandular trichomes we observed in *B. schreberi* represent a simple type consisting of two stalk cells at base and a glandular cell head in plant trichomes (Carpenter, 2006; Huchelmann, Boutry & Hachez, 2017; Tissier, Morgan & Dudareva, 2017). Multicellular trichomes have one or more cells secreting trichome head, stalk cells, intermediate cells, and basal cells, such as peppermint, sunflower and tomato (Turner, Gershenzon & Croteau, 2000; Bergau et al., 2015; Amrehn et al., 2016). A cuticle surrounds the base of the *B. schreberi* glandular trichomes, and is discontinuous with the epidermal surface in aged samples. This epidermal cuticle is similar to that observed in the genus *Genlisea* (Lentibulariaceae), which has a discontinuous cuticle (Plachno, Faber & Jankun, 2005). The discontinuous cuticle is unlike the tight barriers that are in the peripheral mechanical ring, periderm and cuticle—structures that are common in wetland plants along the Yangtze River, such as *Alternanthera philoxeroides* (Yang et al., 2019), *Artemisia lavandulaefolia* (Zhang, Yang & Seago, 2018), *Cynodon dactylon* (Yang et al., 2011), *Phalaris arundinacea* (Zhang et al., 2017a), and *Zizania latifolia* (Yang et al., 2014).

### Ion permeability of glandular trichomes

We used berberine hemisulfate as a permeability trace to assess the ionic permeability of the apoplastic barrier layers of the glandular trichomes. Barriers to absorption typically block berberine from entering inner organs for tests with *Iris* (Meyer, Seago & Peterson, 2009), *Artemisia lavandulaefolia* (Zhang, Yang & Seago, 2018), and *Alternanthera philoxeroides* (Yang et al., 2019). The glandular trichomes of *B. schreberi* secrete mucilage via storage space when young, then transfer that mature stage to be able to absorb greater numbers of ions. Ion permeability ceases at the oldest stages, and in our study Hoechst 33342 staining showed that ions can accumulate both in the cuticle of the storage space and glandular cell. Glandular trichomes are known to secrete mucilage, but this study is the first to observe that trichomes can absorb ions.

### Response of glandular trichomes to stress

It is well known that heavy metal As^3+^, Cd^2+^, and Cr^6+^ poison *B. schreberi* leaves turned yellow and died (Ding et al., 1998; Song, Shi & Yang, 2000; Xu et al., 2000). Hg^2+^, Cd^2+^, and Cr^6+^ can induce organelles to swell, collapse membranes (Li et al., 1998; Yang et al., 2000, 2001; Yang, Shi & Chen, 2001), and deform protoplasts (Ding et al., 1998; Shi et al., 2000) and so cause senescence in glandular cells.

The young glandular cells were more sensitive than mature glandular cells to the NaCl treatments. Nearly half of young glandular cells senesced in the 0.9% treatment, whereas an equal number of mature cells senesced in the 1.8% treatment. Aged glandular cells that senesced showed pillar-like, foam-like and obscure structures. In our ionic permeability test, mature glandular cells absorbed more ions, which may explain why mature glandular cells are more tolerant than young glandular cells to NaCl stress.

Young glandular cells were more tolerant than mature glandular cells to stress with ethanol solutions. Nearly half of young glandular cells senesced under the 3% treatment, whereas nearly half of mature glandular cells had senesced in the 2% treatment at the same time point. Aged glandular cells also showed pillar-like, foam-like, and obscure structures. NaCl and ethanol stress showed that protoplasts have a dynamic, complex organelle membrane system and that young glandular cells are more tolerant to ethanol stress than mature glandular cells. The protoplasts of both young and mature glandular cells appeared to have similar pillar-like, foam-like, and obscure structures in the NaCl and ethanol treatments.

Inorganic and organic ions penetrated the storage space and glandular cell and intensely absorbed the Hoechst 33342 stain in trichomes that were at the mature stage in both the berberine permeability test, and the NaCl and ethanol stress tests. The trichomes cuticles contain osmiophilic material in *Artemisia annua*, *Cannabis sativa*, and *Doronicum* (Mahlberg & Kim, 1991; Duke & Paul, 1993; Muravnik, Kostina & Mosina, 2019). Glandular trichomes are permeable and absorb ions that may contribute to the osmiophilic material in the cuticle of trichomes and dynamic protoplasts. High concentrations of NaCl and ethanol solutions accelerated the senescence of glandular trichomes following just short periods of exposure, which provide new insight that may facilitate fertilizer research (Fu & Wiersema, 2001; Zhang et al., 2015). We suggest that lower concentrations of fertilizer should be used in the water of *B. schreberi* fields.

## Conclusions

Our study revealed that the glandular trichome of *B. schreberi* is composed of stalk cells and a glandular cell surrounding a storage space which is covered with a thin cuticle on the epidermis. The cuticle is discontinuous at the shoots surface. The physiological function of glandular trichomes secrete mucilage via storage space at young stage, then become permeable and absorb ions at the mature stage which depends on the osmiophilic material and the dynamic protoplast, and accelerate senescence and disassembly by ion accumulation. Our analysis of the morphological and physiological characteristics of *B. schreberi* glandular trichomes may facilitate understanding of this endangered plant’s taxonomy, evolution and phylogeny. Permeability tests and ion treatments of glandular trichomes may provide new insight for future fertilizer research (Fu & Wiersema, 2001; Zhang et al., 2015).

## Supplemental Information

10.7717/peerj.7288/supp-1 Supplemental Information 1 Average percentage of young and mature *B schreberi* glandular cells that senesced after treatment with 0%, 0.9%, 1.8% or 2.7% NaCl after 0 min, 10 min, 20 min, and 30 min *in vivo*. in Fig. 5, and with 0%, 1%, 2%, 3% or 4% (v/v) ethanol for 0 min.

10.7717/peerj.7288/supp-2 Supplemental Information 2 *B. schreberi* glandular trichomes on stems and leaves. Scale bars = 50 μm. (A) Storage space (asterisk), glandular cells (arrowhead), mucilage on stems, mature stage, SR7B; (B) Apical view of storage space (asterisk), glandular cells (arrowhead) on petioles, young stage, not stained; (C) Storage space (asterisk), glandular cells (arrowhead) on leaves, young stage, SR7B.

## Additional Information and Declarations

### Data Availability

The following information was supplied regarding data availability: The raw measurements are available in Dataset S1.
