# Supplementary figures and images for: Structure and ion physiology of Brasenia schreberi glandular trichomes in vivo
Source: PeerJ. 2019 Jul 10;7:e7288. doi: 10.7717/peerj.7288 (PMC6802583; doi:10.7717/peerj.7288)

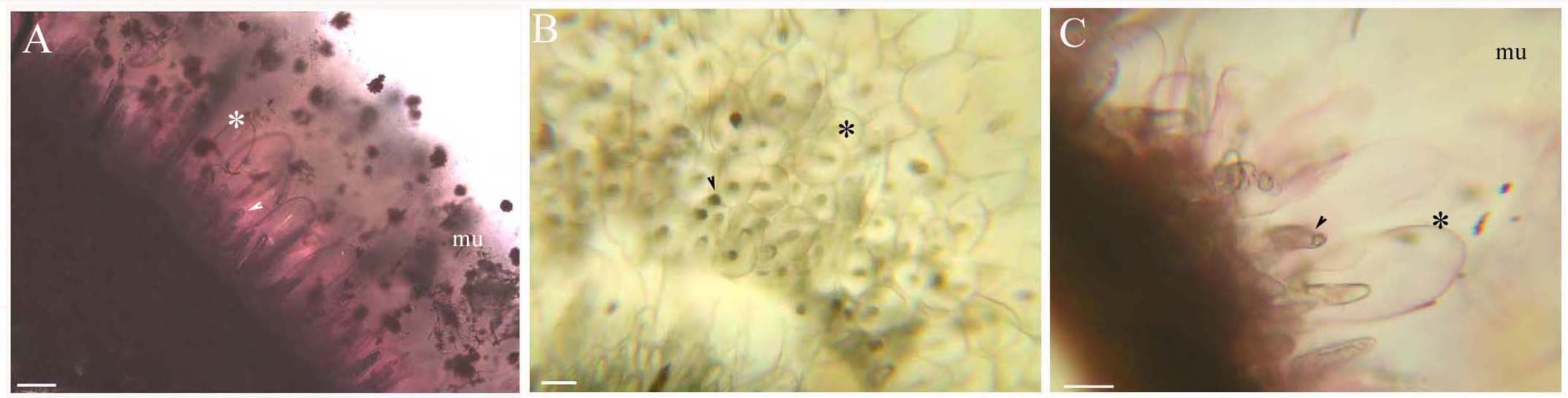

Supplement: Supplemental Information 2 — (A) Storage space (asterisk), glandular cells (arrowhead), mucilage on stems, mature stage, SR7B; (B) Apical view of storage space (asterisk), glandular cells (arrowhead) on petioles, young stage, not stained; (C) Storage space (asterisk), glandular cells (arrowhead) on leaves, young stage, SR7B. [file peerj-07-7288-s002.jpg]
